# How the COVID-19 pandemic shaped Canadians’ preferences for setting of dying: Comparison of two panel surveys

**DOI:** 10.1177/08404704241297037

**Published:** 2024-11-06

**Authors:** Laura M. Funk, Corey S. Mackenzie, Li-Elle Rapaport, Maria Cherba, S. Robin Cohen, Marian Krawczyk, Andrea Rounce, Kelli I. Stajduhar

**Affiliations:** 1 8664 University of Manitoba, Winnipeg, Manitoba, Canada.; 2 6363 University of Ottawa, Ottawa, Ontario, Canada.; 3 5621 Lady Davis Research Institute, Montréal, Québec, Canada.; 4 5620 McGill University, Montréal, Québec, Canada.; 5 3526 University of Glasgow, Glasgow, Scotland, United Kingdom.; 6 8205 University of Victoria, Victoria, British Columbia, Canada.

## Abstract

The purpose of this article is to assess whether COVID-19 shaped Canadians’ preferred settings of dying. We compared data collected using the same survey from two independent but comparable sets of panel respondents, prior to and after the onset of the pandemic. A vignette methodology was used to assess preferences for dying in each of four settings: home, acute/intensive care, palliative care, and long-term residential care. Although preferences for dying at home, in acute/intensive care and palliative care units did not change, preferences for dying in nursing homes significantly declined. In the pandemic’s first and second waves, the spread of knowledge about problems of poor care, visitation restrictions, and fears of contagion in Canadian long-term residential care may have shaped public perceptions of and preferences for dying these settings. If this change persists, it may influence advance care planning decisions. That preferences for dying at home did not shift is noteworthy.

## Introduction

Death at “home,” or at least not in hospital, has been identified as a quality indicator for Canadian healthcare systems.^1^ Although proportions of deaths in hospitals have slowly been declining, this is in part offset not only by increases in home deaths but also deaths in nursing homes (i.e., Long-Term Residential Care or LTRC), as our population ages.^2^ A recent report called for facilitating and promoting home deaths in Canada, which the authors argue aligns with people’s preferences.^3^ Others have likewise documented majority, though by no means universal, preferences to die at home among the broader public.^4,5^

However, people with advanced chronic and terminal illnesses had very limited choice about where they died in the first several waves of the COVID-19 pandemic (i.e., in Canada, roughly January 2020 through 2021).^6^ A population-based study of death certificate data for adults from 32 countries found that home deaths increased in 23 countries during 2020-2021.^7^ In Canada, deaths outside of hospital (including private homes and LTRC) increased by 4% between 2019 and 2020, while hospital deaths decreased by a similar amount (3.9%).^8^ In the United Kingdom, Higginson and colleagues^9^ suggested that increases in home deaths during the pandemic’s early waves were due not only to patient and families’ preferences but also “pandemic-related displacement from healthcare facilities.” Notably, home deaths were often not well supported during this time due to limitations in both home care services and family caregiver supports.^10^

Canada experienced extraordinarily high rates of COVID-19 related deaths in LTRC during the first few pandemic waves. By the end of the first wave, more than 80% of all COVID-19 deaths in Canada had occurred in LTRC facilities. This percentage was significantly higher compared to other Organization for Economic Development (OECD) countries.^11^ Systematic issues such as underfunding, inadequate staffing levels, and outdated infrastructure exacerbated the crisis, revealing long-standing deficiencies in this sector.^12^ The situation became so dire that military troops were at one point deployed to LTRC homes in several provinces to provide support to overwhelmed facilities.

Although pre-pandemic media coverage of LTRC had often been critical,^13,14^ during the pandemic there was also widespread mainstream and social media reporting of poor care conditions, visitation restrictions, contagion, and lone dying of residents in Canada.^15,16^ A high-profile report from the Canadian Armed Forces also detailed disturbing accounts of inadequate care, neglect, and abuse in LTRC in the province of Ontario.^17^

Surveys conducted after the onset of the pandemic, largely focused on Ontario and Québec, have documented public perceptions of poor-quality care^18^ as well as disinclination to enter LTRC facilities.^19^ Other research has suggested the pandemic has generated new fears about dying alone in LTRC^20^ and reinforced perceptions of these as impersonal and medicalized settings.^21^ Indeed, COVID-19 may have shaped Canadians’ preferences for dying in different settings, for instance through fears of contagion, access to visitors, or concerns about care quality. Rigorous longitudinal and national assessment of pandemic-related change in public preferences for setting of dying, however, has been lacking. The objective of the present study is examine two independent but comparable panel survey datasets to assess whether the context of the pandemic affected preferences for dying in particular settings among the Canadian public.

## Methods

Two on-line panel surveys were conducted with different samples prior to and after the onset of the COVID-19 pandemic. These were fielded by Qualtrics using one of their pre-existing panels in Canada. In both iterations of the survey, quotas were used to approximate the Canadian population within respondents based on key sociodemographic variables: age, French as first language, gender, and rural geographic location.

Ethical approvals were received from the first author’s institution and McGill University. Respondents provided informed consent at the beginning of the surveys and chose to complete the measures in either official language of Canada (French and English), on their phone or a computer. Preferences for dying in different settings were assessed the same way in both surveys, using a vignette methodology described more fully elsewhere.^4^ Respondents were asked to imagine that they were currently dying from an illness and presented with three vignettes that represented mild, moderate, and severe situational scenarios based on variation in three aspects: how well pain and other symptoms were managed; availability and access/proximity to formal healthcare services; and availability of informal/family support. In the findings section, therefore, when we refer to “situational severity,” we are referring to the level of severity of the overall vignette, which includes but is not limited to actual symptom severity. After each vignette, respondents were asked to rate separately their preference for each of four settings (with the option to add another): “In this specific situation, how much would you want to spend your last 2 weeks of life in the following places (where 1 is ‘not at all’ and 5 is ‘very much’): your own home or the home of a family member or friend; acute or intensive care unit; a hospice or palliative care setting; nursing home or long-term residential care; another place (please specify).” Thus, the measure acknowledges how such preferences are closely intertwined with people’s preferences for where they would like to be cared for in the last 2 weeks of life.

At T1 (pre-pandemic; August 2019 - January 2020), the final sample of 2,500 represented a 77.2% completion rate, and at T2 (during the pandemic; September 2020), the final sample of 1,009 represented an 89.5% completion rate. The objectives of this analysis were (1) to assess change in preference for place of death between the two time points (for which we used a mixed between and within-subjects analysis of variance); and (2) to assess whether T2 respondents’ preferences were related to their knowledge of and experience with COVID-19.

We addressed our first objective with a mixed between and within-subjects analysis of variance. Time (T1 vs. T2) was the between-subjects factor and the within-subjects factors were preferred place (setting) of dying (home, acute/intensive care, palliative care, and nursing home) and severity of the dying situation (mild, moderate, and severe). Situational severity, place of dying, and time of survey represented independent variables, while preferences for where participants wished to spend their end of life represented the dependent variable. We conducted post-hoc pairwise comparisons using Fisher’s Least Significant Difference (LSD).

To address our second objective of assessing whether T2 respondents’ setting preferences for dying was related to their knowledge of and experience with COVID-19, we analyzed correlations between the COVID-19 variables and setting preferences (situational severity collapsed across care settings).

## Results

Although directly comparable census data is in most cases unavailable, our estimates lead us to conclude that both samples are closely—albeit not perfectly—representative of Canadians 18+ in terms of characteristics such as age, gender, income, religiosity, marital status, and rural or urban residence.^22,23^ We acknowledge slight under-representation of those self-identifying as ethnic minority and over-representation of highly educated respondents in both samples.

Sample comparisons employed Chi-square analyses for categorical variables and t-tests for continuous variables (see Table 1).

**Table 1.** Sample sociodemographic characteristics at T1 and T2.

	Time 1 (%)	Time 2 (%)	Sample comparison
Mean (SD) age	52.3 (17.4)	54.8 (15.7)	*t* (3,501) = −3.98, *P* < .001
Gender	Male: 50.8	Male: 50.1	X^2^ (2) = 4.11, *P* = .250
	Female: 48.8	Female: 49.7	
	Non-binary: 0.5	Non-binary: 0.2	
Language (binary)	English: 77.8	English: 72.6	X^2^ (1) = 11.55, *P* < .001
Relationship status	Separated: 17.7	Separated: 16.1	X^2^ (2) = 7.75, *P* = .021
	Married: 53.8	Married: 59.0	
	Never married: 28.5	Never married: 24.9	
Income (low: 0-59K, med: 60-119K, and high: 120K+)	Low: 51.3	Low: 41.1	X^2^ (2) = 31.34, *P* < .001
	Med: 34.2	Med: 39.9	
	High: 14.5	High: 19.0	
% from large city	73.1	79.0	X^2^ (1) = 12.75, *P* < .001
Ethnicity	White: 81.5	White: 80.3	X^2^ (5) = 10.34, *P* = .066
	Arab: 0.9	Arab: 1.3	
	Asian: 9.6	Asian: 11.1	
	Black: 2.2	Black: 1.3	
	Indigenous: 1.9	Indigenous: 0.9	
	Latino: 1.0	Latino: 1.1	
Canadian citizenship	95.2	96.7	X^2^ (1) = 4.03, *P* = .045
Education	Less than high school diploma: 5.4	Less than high school diploma: 3.4	X^2^ (3) = 25.08, *P* < .001
	Diploma/Some college: 41.2	Diploma/Some college: 34.8	
	University degree: 38.7	University degree: 42.7	
	Graduate study: 14.7	Graduate study: 19.1	
Religious (binary Y/N)	Yes: 62.1	Yes: 61.9	X^2^ (1) = 0.02, *P* = .900

Note. Sample comparisons employed chi-square analyses for categorical variables and t-tests for continuous variables.

The T2 sample was generally comparable to the T1 sample, however age at T2 (M = 54.4; SD = 15.7) was modestly but significantly higher than at T1 (M = 52.3; SE = 17.4). Income and education were also significantly higher at T2. Overall, in addition to being older, the T2 sample was more well educated, had a higher income, and had a larger proportion of French language speakers and a higher proportion of participants living in large cities, than the T1 sample.

Concerning our first objective, there was a significant three-way interaction between place of dying, situation severity, and time of participant preferences for dying (F (6, 3,428) = 3.35, *P* = .003, η_*p*_^2^ = .01). As shown from the means in Table 2, this interaction resulted from a significant reduction in LTRC preference ratings from T1 to T2 in the moderate and severe scenarios. There were no other significant differences (*P* < .01) in preferences for dying in other settings and in other scenarios between participants from T1 to T2.

**Table 2.** Condition desirability means and post-hoc tests.

Place	Severity	Pre-pandemic mean (SE)	During pandemic mean (SE)	Mean difference (SE)	Significance	95% CI for difference	
						Lower	Upper
						bound	bound
1	1	4.39 (.024)	4.33 (.037)	.055	.214	−.032	.141
	2	4.00 (.026)	4.06 (.040)	−.058	.229	−.151	.036
	3	3.04 (.034)	3.10 (.054)	−.059	.356	−.184	.066
2	1	1.82 (.024)	1.83 (.037)	−.005	.909	−.091	.081
	2	2.40 (.026)	2.34 (.041)	.065	.180	−.030	.160
	3	2.98 (.030)	2.94 (.047)	.046	.416	−.064	.156
3	1	2.16 (.026)	2.20 (.042)	−.040	.414	−.137	.056
	2	2.95 (.028)	2.84 (.045)	.110	.037	.006	.214
	3	3.49 (.030)	3.44 (.048)	.050	.377	−.061	.161
4	1	1.76 (.022)	1.70 (.035)	.057	.164	−.023	.138
	2	2.22 (.025)	1.98 (.039)	.244	.000	.153	.335
	3	2.44 (.028)	2.26 (.044)	.178	.001	.077	.280

Note. Place 1 = home, 2 = ICU, 3 = palliative care, 4 = LTRC/nursing home. Severity 1 = mild, 2 = moderate, and 3 = severe. CI = confidence interval. We adopted a *P* < .01 value to indicate significant differences. Means for preference to spend last 2 weeks of life in each setting × situation range from 1 (not at all) to 5 (very much).

As shown in Table 3, the COVID-19 variables measuring greater awareness of death, worries, and adherence to public safety measures were related to greater preferences for hospital-based options of acute/intensive care and palliative care at T2, with correlations ranging from .07 to .15. The COVID-19 variables related to whether the pandemic increased participants’ awareness of death, their worry about self or others, and their attention to public health protocols had no significant correlation with preferences for LTRC however, at T2.

**Table 3.** Zero-order correlations and means (SD) for COVID-19 and place of death preference variables.

Variables	1	2	3	4	5	6	7	8	9	M (SD)
1. Home	--	−.29**	−.40**	−.18**	.08*	.04	.06	.02	−.07*	3.82 (1.03)
2. ICU		--	.57**	.51**	.07*	.09**	.10**	.11**	.07*	2.39 (1.05)
3. Palliative care			--	.46**	.02	.07*	.08**	.11**	.15**	2.86 (1.15)
4. Long-term residential care				--	.03	.05	.05	.04	−.02	2.09 (1.03)
5. COVID raised awareness of death					--	.58**	.52**	.52**	.17**	2.84 (1.25)
6. Worry about getting COVID						--	.69**	.70**	.26**	3.40 (1.34)
7. Worry passing COVID to at-risk family							--	.82**	.30**	3.41 (1.27)
8. Worry getting family ill from COVID								--	.36**	3.52 (1.16)
9. Follow COVID public health guidelines									--	4.53 (.82)

Note. Each item is rated on a 5-point scale ranging from 1 (not at all) to 5 (very much). *P < .05, **P < .01.

## Discussion

We surveyed generally comparable and representative panel respondent samples just prior to the onset of the pandemic and during its second wave, using the same survey tool. Although preferences for dying at home, in acute/intensive care and in palliative care/hospice did not change, there was a significant decline in adult Canadians’ preferences for dying in LTRC. The spread of knowledge and concern about problems of poor care, visitation restrictions, and fears of contagion in Canadian LTRC settings may have been influential in this regard. People may also have had first-hand experience with a family member or friend in LTRC during this time, or have known or been someone working in healthcare, especially LTRC.

These findings support international research on the eroding trust in LTRC since the pandemic, demonstrating public aversions to not only living but also dying in these settings.^20,21,24^ Achou and colleagues^19^ suggest LTRC disinclination may mean that people may be more likely to save for their older age and/or to support public policies subsidizing home-based care and supports (see also Amilon et al.^25^). Ultimately, however, such aversion, for those who lack access to appropriate home care resources (including palliative care), can exacerbate difficult decisions and subsequent challenges for families. Fear, distress, vigilance, guilt and shame may be amplified for those who have no other choice but LTRC.

Public preferences for dying at home did not discernably shift in the present study. Dying at home during a pandemic may be less desirable as it may have been simultaneously less supported and less of a choice during this time,^26^ especially for those without family support. That public preferences for dying at home did not increase also diverges from the perspectives of policy and practice stakeholders we interviewed for our study.^10^ However, those stakeholders may have been inferring preferences from overall shifts in settings of death; they were also contemplating patient preferences, whereas the survey assessed the general population. Indeed, preferences may have increased for dying at home among patients with advanced illnesses, or for families with direct experience with LTRC during the pandemic, rather than among the public.

The lack of a correlation between COVID-19-related measures (awareness of death, worries, and adherence to public safety measures) and respondents LTRC preferences at T2 may have been because individuals had such low preferences for that option overall (i.e., a basement effect). We did, however, find small, positive correlations between higher responses to these questions (i.e., greater awareness/concern) and greater preferences for spending either the last 2 weeks of life in a hospital/ICU or palliative care units. Respondents may have believed they would have better access to adequate medical or other kinds of care in these settings during this time in which such access was particularly problematic either in one’s home or in LTRC.

Future studies should address whether and how COVID-19 has shaped preferences among people with advanced and terminal conditions and assess whether a public disinclination for LTRC settings persists as the pandemic and restrictions have abated, especially as significant staffing and resource issues persist in Canada. Moreover, further research should explore how perceptions of and trust in LTRC are intertwined with trust in other social systems,^27^ and among persons from groups more likely to be harmed within institutional settings (e.g., LGBTQ persons, racialized persons, and those with advanced dementia). Importantly, our samples did not fully reflect the cultural and racial diversity of the Canadian population, limiting our analytic ability to explore these influences in our data.

Our previous analyses of the T1 dataset illuminated sources of micro-level contextual variation that shape peoples’ preferences for dying in different settings, including age and attitudinal support for family obligations.^4^ Our qualitative research has also highlighted how preferences for dying at home can be tempered or muted in the context of inadequate services or infrastructure (such as housing) or concerns about impacts on family; moreover, that preferences for dying at home do not necessarily represent choices between viable options for those facing structural vulnerabilities.^28,29^ Indeed further research should also more fully explore how both local and regional variations in access to services and publicly funded supports, as well as variations in peoples’ social locations (e.g., socio-economic status) and cultural background can shape these perceptions.

The present analyses demonstrate that COVID-19 may have had an impact on Canadian public preference about LTRC as an acceptable place to die, illuminating the importance of contextual influences on public preferences for settings of dying. The sensitivity and appropriateness of advance care planning processes can be enhanced if providers receive education that helps them recognize and respond to the complexity and relationality of these processes, and to understand how social contexts can shape peoples’ feelings and perceptions about different settings of care over time.^10,30^

For policy-makers, the findings raise questions about how future preferences for settings of end-of-life care and death may shift depending on governmental and other forms of collective action in the years ahead. In particular, structural changes in the LTRC sector could enhance working conditions and address the workforce crisis, while promoting person- and family-centred care and localized, relational approaches to problem solving.^31,32^ If such changes occur and become known to the public, perceptions of and trust in LTRC may improve. Policy-makers could enhance transparency in the sector through regulatory frameworks and government direction aimed at structural change, and by providing resources to address residents’ social isolation. Policies to support higher wages and better working conditions can increase confidence in appropriate care practices in the sector. Engaging LTRC providers/operators in strategic reflection and planning should prioritize person- and family-centred care. Into the future, comprehensive system changes should also include LTRC staff in shared governance and collaborative policy development.

Ultimately, whether our findings support significant improvements in LTRC working and care conditions^32^ or growing calls for further deinstitutionalization^33^ is a matter of perspective, although in our broader work we have argued for a need to critically explore public assumptions associated with, and implications of, dying at home.^28,29^ Indeed, resourcing to support quality care and dying well both in LTRC and home settings may be imperative, including investments in palliative care in these settings.^34^ Ultimately, regardless of public preferences, policy emphases and actual increases in home death, numerous studies have identified that LTRC will remain a critical setting for the end of life and dying in Canada and beyond.^35^ As a result, the complex forces shaping perceptions of dying in these settings, as well as perceptions of and trust in formal home-based and palliative care services, remains an imperative for future research.

## Data Availability

The datasets generated and/or analyzed during the current study are not yet publicly available as they are still be used for additional primary analyses and publications by the research team but are available from the corresponding author on reasonable request.
